# Par6b Regulates the Dynamics of Apicobasal Polarity during Development of the Stratified *Xenopus* Epidermis

**DOI:** 10.1371/journal.pone.0076854

**Published:** 2013-10-18

**Authors:** Sha Wang, Sang-Wook Cha, Aaron M. Zorn, Christopher Wylie

**Affiliations:** 1 Division of Developmental Biology, Cincinnati Children’s Research Foundation, Cincinnati, Ohio, United States of America; 2 Molecular and Developmental Biology Graduate Program, Cincinnati Children’s Research Foundation, Cincinnati, Ohio, United States of America; Texas A&M International University, United States of America

## Abstract

During early vertebrate development, epithelial cells establish and maintain apicobasal polarity, failure of which can cause developmental defects or cancer metastasis. This process has been mostly studied in simple epithelia that have only one layer of cells, but is poorly understood in stratified epithelia. In this paper we address the role of the polarity protein Partitioning defective-6 homolog beta (Par6b) in the developing stratified epidermis of *Xenopus laevis*. At the blastula stage, animal blastomeres divide perpendicularly to the apicobasal axis to generate partially polarized superficial cells and non-polarized deep cells. Both cell populations modify their apicobasal polarity during the gastrula stage, before differentiating into the superficial and deep layers of epidermis. Early differentiation of the epidermis is normal in Par6b-depleted embryos; however, epidermal cells dissociate and detach from embryos at the tailbud stage. Par6b-depleted epidermal cells exhibit a significant reduction in basolaterally localized E-cadherin. Examination of the apical marker Crumbs homolog 3 (Crb3) and the basolateral marker Lethal giant larvae 2 (Lgl2) after Par6b depletion reveals that Par6b cell-autonomously regulates the dynamics of apicobasal polarity in both superficial and deep epidermal layers. Par6b is required to maintain the “basolateral” state in both epidermal layers, which explains the reduction of basolateral adhesion complexes and epidermal cells shedding.

## Introduction

Epithelial apicobasal polarity plays important roles in morphogenesis during embryogenesis [1], and is lost or dis-regulated in many disease processes, including metastatic carcinomas [2], [3], [4]. Apicobasal polarity has mostly been characterized in simple epithelia, in which cells are arranged in a single layer with clear apical and basolateral membrane domains separated by sub-apical junctions. However, many epithelial tissues, such as the epidermis, are composed of multiple layers, known as stratified epithelia. The superficial layer has a contact-free apical surface, while all membranes of deep layer cells are in contact with other cells. So the polarity status likely varies in different layers. Little is known about what regulates apicobasal polarity in stratified epithelia [5], especially in deep layer cells. In monolayered *C. elegans*, *Drosophila* and cultured mammalian epithelial cells, key polarity proteins Par6, aPKC and Crb are apically localized [6], [7], [8]. However, in the newborn stratified mouse skin, aPKC is localized in basal keratinocytes at the dermal/epidermal junction, Par6 is enriched in the cytoplasm of the granular layer, and Crb3 exhibits a diffuse pattern at the basal side of basal cells [9]. The different distributions of conserved polarity proteins and different architecture between simple and stratified epithelia raise the question of how the core polarity machinery acts in stratified epithelia.

Three evolutionarily conserved protein complexes guide the polarization process: Crb, Par and Scribble. Antagonistic interactions between these three complexes, mediated by protein-protein interaction and protein phosphorylation, are thought to segregate the apical and basolateral membrane domains [10], [11], [12]. The Par complex was first identified as essential for asymmetric cell division in the *C. elegans* zygote [13], [14], [15], and comprises Par6, Partitioning defective-3 (Par3), aPKC and activated Cdc42. Par6 serves as the scaffolding protein in the Par complex. It contains an N-terminal Phox and Bem1 (PB1) domains, a semi-Cdc42/Rac interactive binding (semi-CRIB) domain, and a C-terminal PDZ domain. Par6 binds to aPKC via the PB1 domain and to Par3 through the PDZ domain [16], [17]. Par6 can also interact with components of both Crb and Scribble complexes by its PDZ domain, which allows functional cross talk between these complexes [18], [19]. The Crb complex, containing Crb, PALS1 and PATJ, defines the apical membrane domain. The Scribble complex, containing Lgl, Dlg, Scribble, establishes the basolateral membrane domain. Crb marks apical membranes [20], [21], [22], [23], [24], whilst Lgl2 localizes to basolateral membranes [23], [24], [25], [26] in *Drosophila* epithelia, cultured mammalian epithelial cells and *Xenopus* blastula presumptive epithelia. Crb and Lgl are conserved apical and basolateral membrane markers, respectively.

Par6 acts as a cornerstone of apicobasal polarity and regulates the delicate balance between apical and basolateral membrane domains [4], [10]. However, it is not known whether Par6 acts primarily to reinforce apical or basolateral identity. In *Drosophila*, when both maternal and zygotic pools of Par6 are removed, epithelial cells lose their laminar organization and apical proteins fail to localize [7]. Zebrafish studies show that in *par6gamma-b* mutant embryos, the neural tube lacks continuous apical membranes and has multiple lumens [27]. Mammals have three Par6 isoforms: Par6alpha, Par6beta and Par6gamma, each with different subcellular localizations and distinct effects on tight junction (TJ) assembly in MDCK cells, indicating that Par6 isoforms may function differently [28]. However mouse mutant phenotypes have not been described. In *Xenopus*, Par6b has been shown required for normal neural tube closure [29], but its impact on apicobasal polarity in this context was not examined.

Here we use *Xenopus* embryonic epidermis as an *in vivo* model to understand how stratified epithelium becomes polarized in development and to determine the role of Par6b in this process. We focus on two representative developmental stages, the late blastula stage (st9), when non-neural ectoderm is undifferentiated (presumptive) epidermis, and the neurula stage (st17), when non-neural ectoderm becomes differentiated epidermis. We first show that superficial and deep ectodermal cells exhibit different distribution of apicobasal polarity markers between the blastula and neurula stages, indicating a dynamic polarity remodeling process. Second, we confirm that *par6b* is expressed in all layers of non-neural ectoderm and show that Par6b depletion in the epidermis by a Par6b antisense morpholino oligo (Par6b-MO) [29] causes epidermal cell dissociation at the tailbud stage. This defect is rescued by subsequent injection of MO-resistant *par6b* mRNA, indicating a specific loss-of-function phenotype. The basolateral adherens junction component E-cadherin is dramatically reduced after Par6b depletion. Third, we show that in normal development the apical marker Crb3 is localized to cytoplasmic vesicles in deep epidermal cells. Par6b depletion reverses this situation, resulting in stabilization of Crb3 to the entire surface of deep cells. Par6b depletion destabilizes Lgl2 in both epidermal layers. In summary, Par6b is required for both the apicobasal polarity and integrity of the stratified *Xenopus* embryonic epidermis.

## Results

### 1. Both Superficial and Deep Ectodermal Cells Acquire New Apicobasal Polarity during Gastrulation

**Figure 1:**
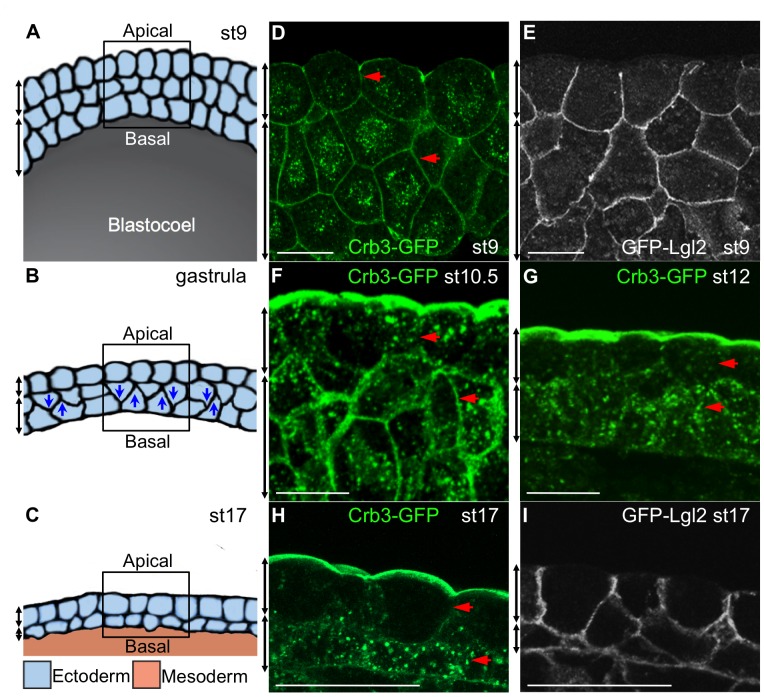
The dynamics of Crb3 and Lgl2 subcellular localization in the developing *Xenopus* stratified epidermis. (**A, B, C**) Schematic representations of the epidermis development at blastula (st9), gastrula and neurula (st17) stages, respectively. Blue arrowheads in panel B illustrate the radial interdigitation of deep cells in the gastrula ectoderm. The black boxes mark the imaging area with the apical surface facing up. (**D, F, G, H**) Crb3-GFP (green) distribution in st9, 10.5, 12, 17 ectodermal cells, respectively. Red arrowheads indicate dynamic subcellular changes of Crb3-GFP. (**E, I**) GFP-Lgl2 (white) distribution in st9 and st17 ectodermal cells. (**A–I**) Double-headed black arrows on the left side of each panel show the boundaries of the superficial and deep layers. (**D–I**) Scale bars, 50 µm.

The *Xenopus* embryonic epidermis is a stratified epithelium that originates from the ventral part of the blastocoel roof at the blastula stage. In the blastula this presumptive epidermis contains one superficial layer and two to three deep layers (Fig. 1A). During gastrulation, deep cells are rearranged into one layer by radial interdigitation [30], [31] (Fig. 1B) and become the basal layer of the epidermis [32] (Fig. 1C). It has been reported that the superficial presumptive epidermal cells are polarized whilst deep presumptive epidermal cells are non-polarized at the blastula stage [23], [33]. As the epidermis differentiates, the superficial cells become morphologically polarized along the apicobasal axis at st12–13 based on the distribution of cell contents such as yolk platelets and mitochondria [32]. To date an analysis of polarity in the deep cells of the differentiating epidermis has not been reported.

To characterize the apicobasal polarity of the developing stratified epidermis, we examined subcellular distributions of the apical membrane marker Crb3 and the basolateral marker Lgl2 during development. Since antibodies to endogenous Crb3 and Lgl2 are not available, we injected mRNA encoding GFP-tagged Crb3 (75 pg) or GFP-tagged Lgl2 (100 pg) into oocytes respectively. After 5 hrs culture allowing mRNAs to diffuse throughout the oocyte cytoplasm, oocytes were matured *in vitro* and fertilized by the host transfer technique [34]. This dose of *crb3-gfp* or *gfp-lgl2* injection had no detectable impact on epidermis development but enabled us to monitor their subcellular localization.

The distributions of Crb3-GFP and GFP-Lgl2 proteins were analyzed at the late blastula (st9) and neurula (st17) stages. In superficial layer cells of the blastula ectoderm, Crb3-GFP was localized to both apical and basolateral membranes (Fig. 1D), whilst GFP-lgl2 was localized only to basolateral membrane (Fig. 1E), as previously reported [23]. This incomplete separation between Crb3 and Lgl2 suggested that superficial cells at this stage were partially polarized. In contrast, in deep cells of the blastula ectoderm, Crb3-GFP and GFP-Lgl2 were both evenly distributed all around the membrane (Fig. 1D, E), indicating that deep presumptive epidermal cells were not polarized. By the neurula stage (st17), the subcellular pattern of Crb3-GFP had changed dramatically. In superficial epidermal cells, Crb3-GFP disappeared from the basolateral membrane and became apically restricted (Fig. 1H), and GFP-Lgl2 maintained on the basolateral membrane (Fig. 1I), indicating that superficial cells become fully polarized at this stage. In deep epidermal cells, Crb3-GFP was lost from the surface membrane and became localized to cytoplasmic punctae (Fig. 1H). In contrast, GFP-Lgl2 remained on entire membranes (Fig. 1I). This loss of membrane Crb3 but not Lgl2 suggests that deep cells membranes have acquired the “basolateral” identity. We next examined intermediate time points to determine when in development these changes occurred. By the early gastrula (st10.5), Crb3-GFP had disappeared from basolateral membranes of the superficial cells and was becoming enriched on apical membranes (Fig. 1F). Between st10.5 and st12, membrane Crb3 in the deep cells progressively redistributed to punctae in the cytoplasm (Fig. 1G). We conclude that apicobasal polarity in both superficial and deep layers changes dynamically during the gastrula stage.

### 2. The Subcellular Localization of Par6b Changes in *Xenopus* Epidermis during Development

**Figure 2:**
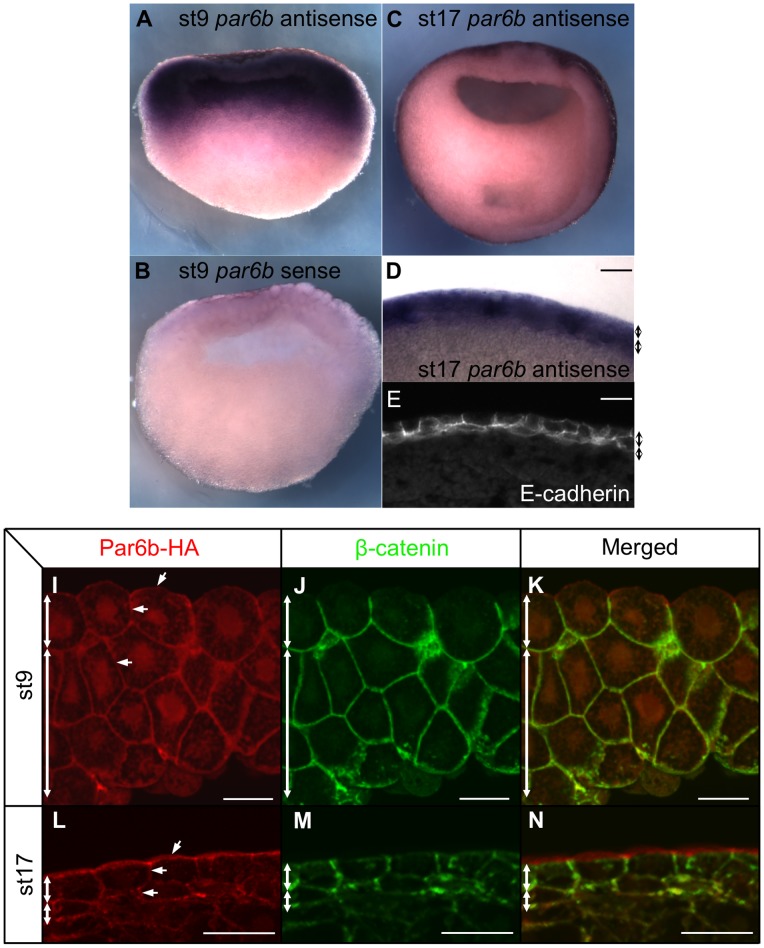
The expression pattern of *par6b* mRNA and the subcellular localization of Par6b protein during development. (**A**) Sagittal view of *par6b* expression pattern in a st9 blastula embryo by whole mount ISH. (**B**) Sense *par6b* probe control in a st9 embryo. (**C**) Transverse view of *par6b* expression pattern in a st17 neurula embryo. (**D**) *par6b* expression pattern in a transverse section of st17 embryo. (**E**) Staining of E-cadherin in the epidermis in a transverse section of st17 embryo. (**I–N**) Staining with anti-HA of Par6b (red) and anti-β-catenin (green) antibodies on st9 or st17 ectoderm. (**D–N**). Double-headed arrows (black in panel D and E, white in panel I–N) respectively indicate boundaries of the superficial and deep layers. Scale bars, 50 µm.

Par6 has been reported to regulate apicobasal polarity in simple epithelia but has not been explored in stratified epithelia. We aimed to determine whether Par6b played a role in the polarity remodeling process that occurred in both layers of the developing epidermis. We first examined the *par6b* expression pattern in detail by *in situ* hybridization (ISH). In oocytes, maternal *par6b* mRNA was localized to the animal hemisphere (Fig. S1A). At the blastula stage, *par6b* mRNA was still enriched in the animal hemisphere (Fig. 2A, S1B, S1B′), the ventral part of which was the presumptive epidermis. By mid-gastrula, *par6b* was expressed in all layers of ectoderm cells including bottle cells (Fig. S1C, D, D′). At st17, *par6b* transcripts were detected in all layers of the epidermis (Fig. 2C, D), as determined by comparison to E-cadherin staining in sibling embryos (Fig. 2E). In early tailbud embryos, *par6b* remained highly expressed in both layers of the differentiated epidermis, as well as in the eyes and cement gland (Fig. S1E, F, F′). Overall, the *par6b* expression pattern supports its potential involvement in the dynamic polarity remodeling process.

In the absence of a specific antibody to detect endogenous *Xenopus* Par6b, we used a mRNA encoding HA-tagged *Xl* Par6b to examine its subcellular localization in embryonic epidermis. 100 pg of *par6b-HA* mRNA was injected into oocytes. These were cultured for 5 hrs to allow diffusion of the mRNA throughout oocytes, then fertilized and collected at st9 and st17. This dose of *par6b-HA* had no detectable impact on development, but anti-HA immunostaining allowed us to examine the subcellular localization of Par6b in comparison to co-staining for beta-catenin, which was localized to basolateral membranes of superficial cells and all membranes of deep cells (Fig. 2J, M). At st9, Par6b-HA protein was evenly distributed all around membranes and also showed diffuse cytoplasmic and nuclear staining in both superficial and deep cells (Fig. 2I, K). This nuclear and cytoplasmic Par6b pattern is consistent with reports from mammalian epithelial cells [35]. However, at st17, Par6b-HA was found exclusively on plasma membranes. It was evenly distributed to the entire membranes of the deep epidermal cells, but more apically in superficial epidermal cells (Fig. 2L, N). This dynamic subcellular localization suggests that Par6b might play an initiating role in the polarization of the epidermis.

### 3. Par6b Depletion Causes Loss of E-cadherin Expression and Epidermal Cell Dissociation at Tailbud Stage

**Figure 3:**
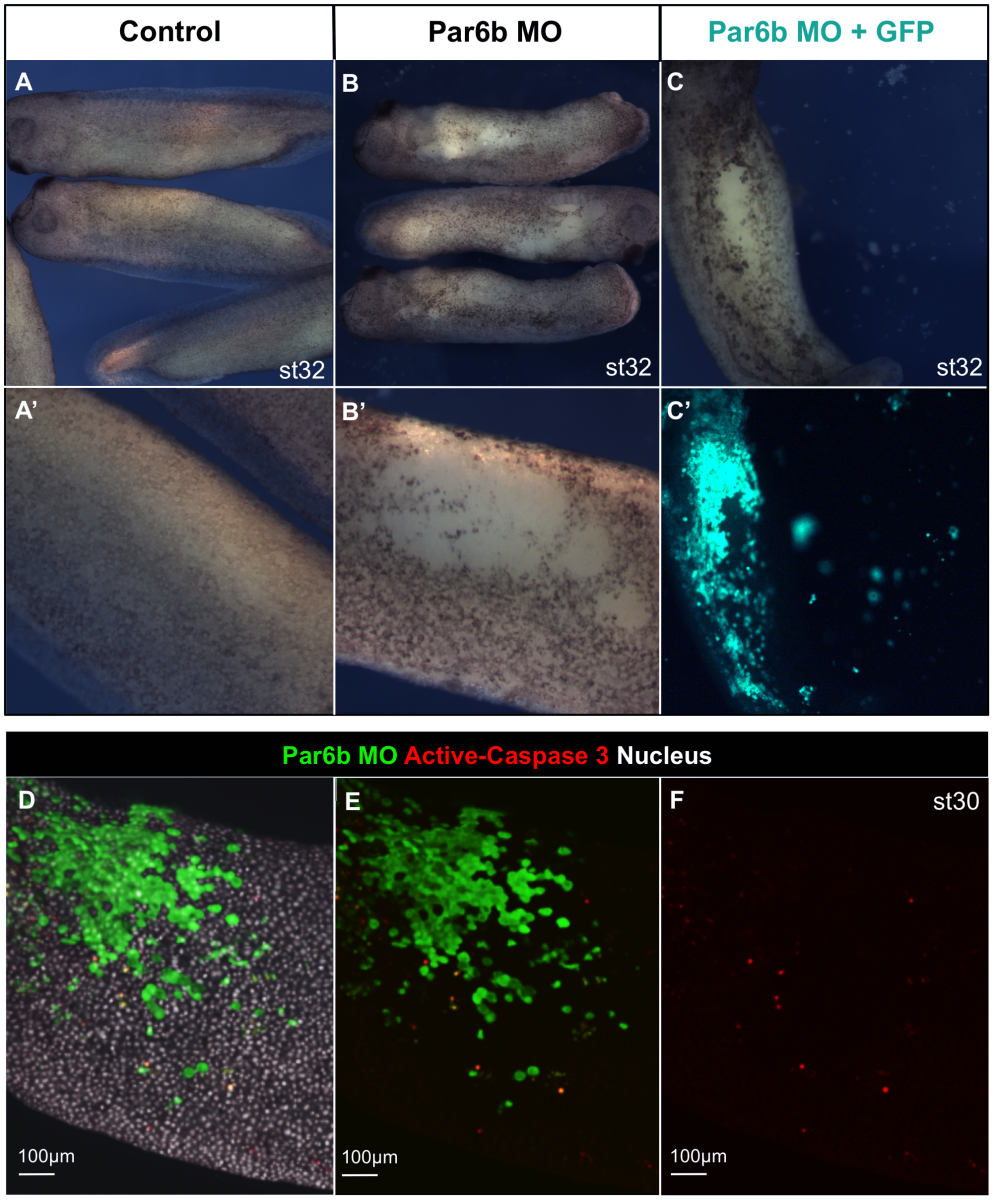
Par6b depletion causes epidermal cell dissociation. (**A, A’**) st32 uninjected embryos and magnified image of the epidermis. (**B, B’**) st32 embryos injected with Par6b-MO into the two animal ventral blastomeres at the 8-cell stage and corresponding magnified image of the epidermis. (**C, C’**) st32 embryos were injected with Par6b-MO and GFP mRNA into two animal ventral blastomeres at the 8-cell stage. Par6b-MO injected (GFP positive) epidermal cells were shed into petri dish. (**D–F**) 5 ng of Par6b-MO was co-injected with GFP into one animal ventral blastomere at 16-cell stage. Whole-amount staining of activated-Caspase 3 (Red), nucleus (white) and GFP (Green) indicated that the number of Activated-Caspase 3 positive cells was not increased in Par6b-depleted clones (GFP positive) at st30.

To understand the role of Par6b in the developing epidermis, we injected a previously validated translation-blocking morpholino oligo against Par6b (Par6b-MO) [29], at doses of 10 ng/cell, into two ventral animal blastomeres (which give rise to the epidermis) at the 8-cell stage. Early development of the embryos was morphologically normal. However, epidermal cells started to dissociate around st32 (late tailbud) and were subsequently shed (Fig. 3A–B′). We then co-injected Par6b-MO with GFP mRNA as a lineage tracer into two ventral animal blastomeres at the 8-cell stage. Par6b depleted skin cells were GFP positive and finally detached from embryos and fell on petri dish (Fig. 3C, C′). To verify whether this was caused by apoptosis, we analyzed the apoptosis marker activated Caspase-3 at stage 30, immediately before skin cell sloughing. Immunofluorescence for activated Caspase-3 showed no difference between control and Par6b-depleted cells in the epidermis (Fig. 3D–F), indicating that cell sloughing was not due to elevated apoptosis.

**Figure 4:**
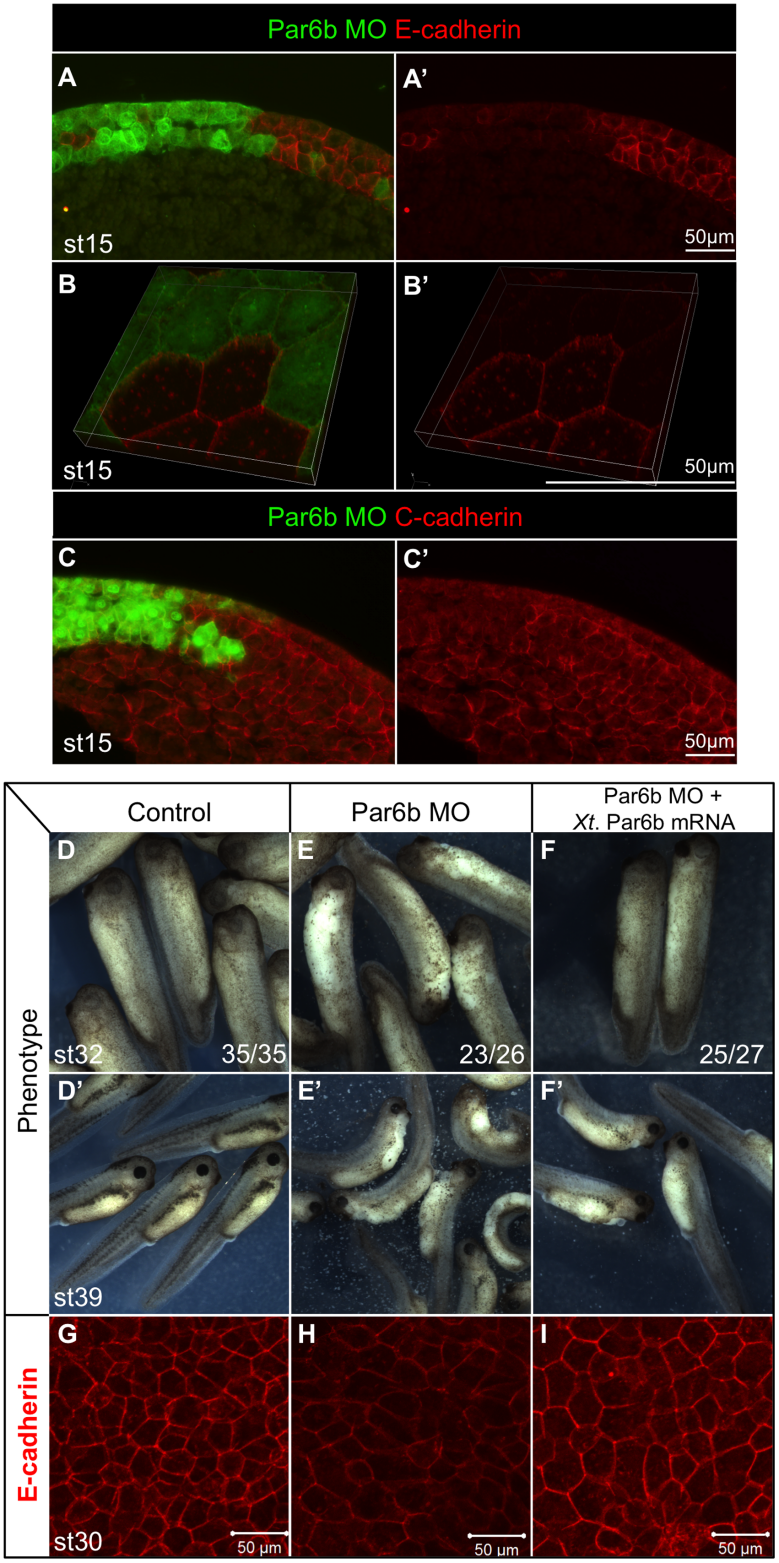
Par6b depletion reduces epidermal cadherins. (**A–C’**) Embryos were injected with Par6b-MO together with GFP mRNA into one animal ventral blastomere at the 8-cell stage. Staining of E-cadherin (A, A’) or C-cadherin (C, C’) (red) and GFP (green) in sections of st15 embryos. Surface magnified view of whole-mount staining of E-cadherin and GFP of st15 injected embryos (B, B’). (**D–F’**) Phenotypes of uninjected, Par6b-MO injected alone, and Par6b-MO with *Xt par6b* mRNA injected embryos at st32 and st39. (**G,** **H, I**) Corresponding whole-mount staining of E-cadherin on st30 epidermis.

This raised the possibility that Par6b disrupts either the expression or function of adhesion molecules that hold epidermal cells together. To test this possibility we performed a mosaic analysis co-injecting 10 ng of Par6b-MO with GFP mRNA as a lineage tracer into a single ventral animal blastomere at the 8-cell stage, so that clones of Par6b-depleted cells lie next to uninjected cells. Immunostaining for adherens junction proteins E-cadherin and C-cadherin at the mid-neurula (st15), prior to epidermal dissociation, revealed that E-cadherin levels were dramatically reduced in Par6b-depleted cells in both superficial and deep epidermal layers (Fig. 4A, A′). Higher magnification of whole-mount E-cadherin staining from the ectoderm surface clearly shows that E-cadherin was cell-autonomously reduced (Fig. 4B, B′). C-cadherin was also reduced in Par6b-deficient cells, but to a lesser extent than E-cadherin (Fig. 4C, C′). Control-MO injection did not alter the levels of E- and C-cadherin (Fig. S2). In addition, cadherin-associated catenins (alpha, beta and gamma), beta1 integrin as well as the tight junction protein ZO-1 [29], were also reduced in Par6b-depleted cells to varying degrees (Fig. S3A–D, F). In contrast there was little if any change in the immunostaining levels of alpha5 integrin. The remaining adhesion molecules C-cadherin and alpha 5 Integrin (Fig. S3E) might explain why Par6b-depleted epidermis, which lacks E-cadherin, did not dissociate until later stages when C-cadherin expression is normally turned off [36]. To confirm the specificity of the Par6b-MO effect, we injected 10 ng of Par6b-MO either alone or sequentially with 300 pg of MO-resistant *Xt par6b* mRNA into two ventral animal blastomeres at the 8-cell stage. *Xt par6b* mRNA rescued both epidermal cell dissociation (Fig. 4D–F′) and E-cadherin levels (Fig. 4G, H, I), demonstrating that these phenotypes were specifically due to the loss of Par6b protein.

### 4. Par6b Depletion does not Change Ectodermal Cell Fate

Par proteins are involved in cell fate maintenance in worms and flies [37], [38]. The reduction of epidermal E-cadherin by Par6b depletion could be due to a prior requirement for Par6b in ectoderm specification. To test this possibility, we used qPCR to assay mRNA levels of several early ectodermal markers, including *xk81a1* (*epidermal keratin*) [39], *sox2* [40] and *cdh2* (*n-cadherin*) [41] in Par6b-depleted embryos at different development stages. None of these markers were affected by Par6b depletion (Fig. S4A). Moreover, immunostaining with cytokeratin antibody, CK7 [42], demonstrated that Par6b-depleted ectodermal cells still expressed cytokeratin protein (Fig. S4B). We conclude that Par6b is not required for early specification of ectoderm.

### 5. Par6b Depletion Reduces E-cadherin Protein Level Post-transcriptionally

**Figure 5:**
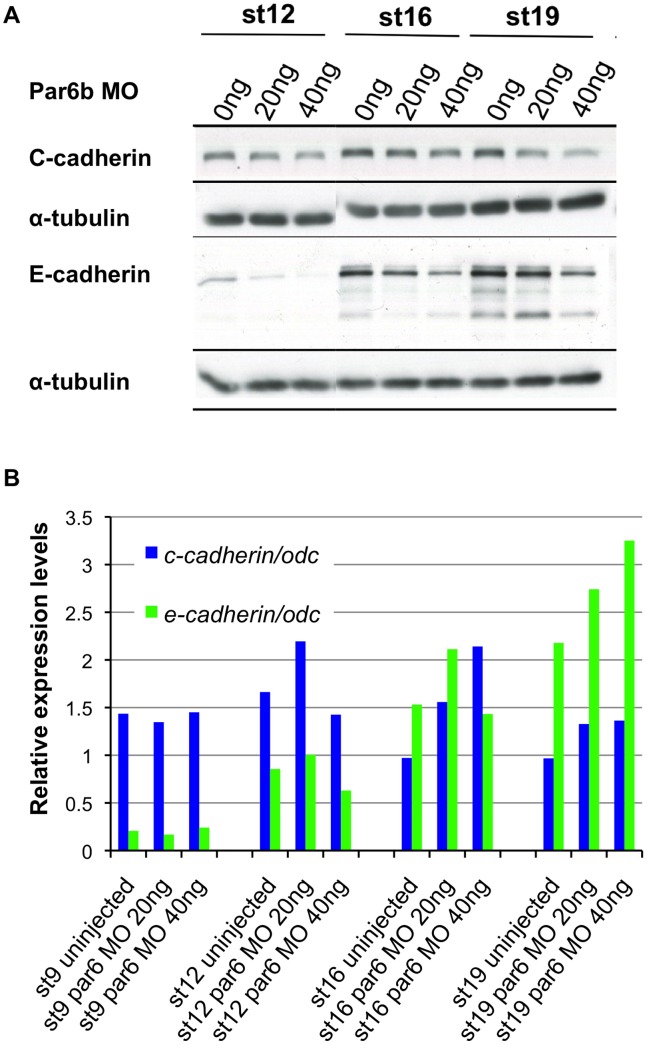
Par6b depletion reduces protein levels but not the mRNA levels of E- and C-cadherin. (**A**) Western blot analysis of total protein extracts from uninjected, 20 ng and 40 ng Par6b-MO injected embryos with anti-C-cadherin and E-cadherin antibodies. (**B**) Quantitative RT-PCR results of *e-cadherin* and *c-cadherin* mRNA levels by two doses of Par6b-MO injection at st9, 12, 16 and 19.

Loss of membrane-localized E-cadherin by Par6b depletion could be due either to lower *e-cadherin* mRNA level, reduced protein level, or failure of membrane presentation. Total E-cadherin protein levels were assayed by western blotting of st12, st16 and st19 embryos, which were injected with 20 ng and 40 ng of Par6b-MO at the two-cell stage. Par6b depletion reduced the total amount of E-cadherin protein in a dose-dependent fashion (Fig. 5A). However qPCR results showed that Par6b depletion had no effect on *e-cadherin* mRNA levels (Fig. 5B). Similarly the total protein but not mRNA level of C-cadherin was also modestly reduced by Par6b depletion (Fig. 5A, B). These data suggest that Par6b regulates the level of E-cadherin protein post-transcriptionally.

### 6. Depletion of Par6b Stabilizes Membrane Crb3 in Deep Epidermal Cells, and Destabilizes Membrane Lgl2 in Both Superficial and Deep Epidermal Cells

**Figure 6:**
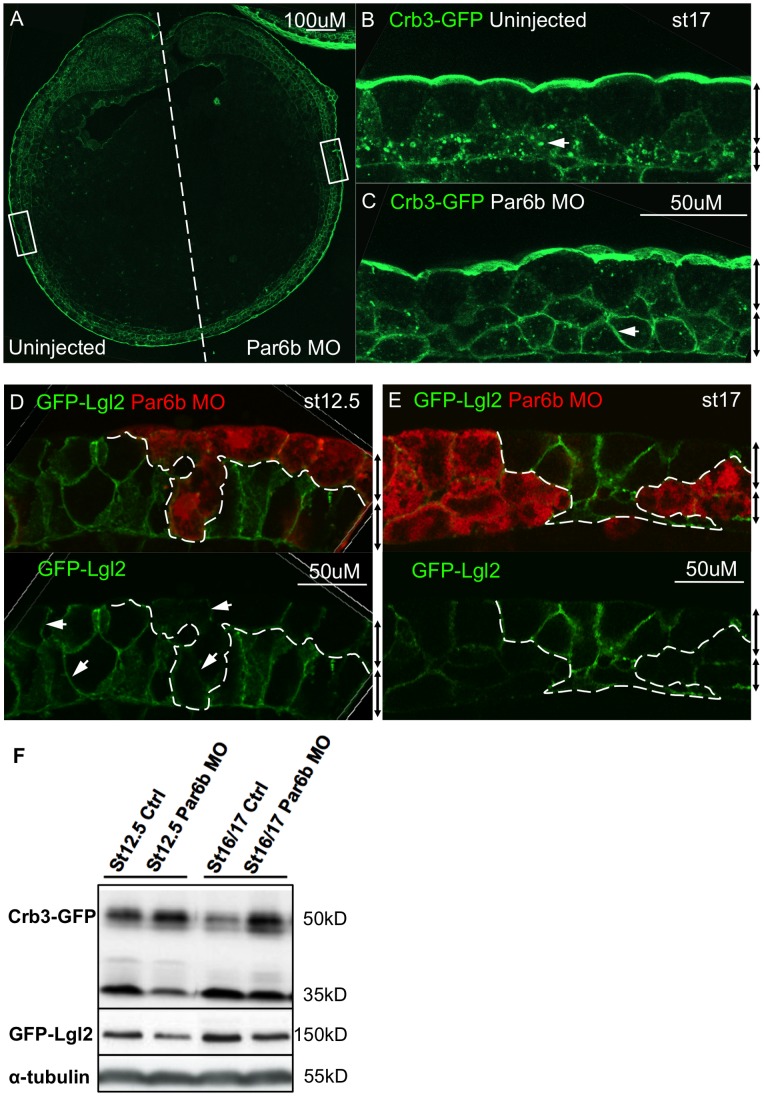
Par6b depletion stabilizes Crb3 on deep cell membranes, and destabilizes Lgl2 in both epidermal layers. (**A**) Crb3-GFP signal in a transverse section of st17 embryos. 25 ng of Par6b-MO was injected into one blastomere at the 2-cell stage. Left half is the uninjected side and right half is the Par6b-MO injected side. (**B**) Crb3-GFP distribution in uninjected epidermis, left box in figure A. (**C**) Crb3-GFP distribution in Par6b-depleted side, right box in figure A. (**D, E**) GFP-Lgl2 signal in transverse section of st12.5 and st17 injected embryos. 10 ng of Par6b-MO with RIDX was injected into one animal ventral blastomere at the 8-cell stage. (**E**) Western blot of total protein extracts from st12.5 and st16/17 Crb3-GFP or GFP-Lgl2 injected host transfer embryos with or without injection of 50 ng Par6b-MO.

To test the hypothesis that Par6b regulates apicobasal polarity of the developing epidermis, we assayed the subcellular distribution of the apical membrane marker Crb3 and basolateral marker Lgl2 after Par6b depletion. A subphenotypic dose of *crb3-gfp* mRNA (75 pg) was injected into cultured full-grown oocytes, which were then fertilized by host transfer. At the 2-cell stage, 25 ng of Par6b-MO was injected into one blastomere, which gives rise to half of the embryo, and the subcellular localization of Crb3-GFP was assayed at st17 on both sides (Fig. 6A). We observed that in the deep epidermal cells on the control side Crb3-GFP was localized in typical cytoplasmic punctae as expected (Fig. 6B). Interestingly, on Par6b-depleted side of the embryo, Crb3-GFP was lost from the cytoplasm and highly enriched on the deep epidermal cell membranes (Fig. 6C). In contrast, there was no obvious change of the apically localized Crb3-GFP in superficial cells by Par6b depletion (Fig. 6B, C). Similarly, the distribution of the basolateral membrane marker Lgl2 was also assayed by injecting 10 ng of Par6b-MO together with rhodamine dextran (RIDX) lineage tracer into one animal ventral blastomere of *gfp-lgl2* injected host transfer embryos at the 8-cell stage. Although GFP-Lgl2 subcellular distribution pattern was unchanged after Par6b depletion, the overall levels of GFP-Lgl2 fluorescence was cell-autonomously reduced in both superficial and deep epidermal cells at st12.5 (Fig. 6D) and st17 (Fig. 6E).

As Par6b regulates cadherin levels post-transcriptionally, we also examined the total protein level of Crb3-GFP and GFP-Lgl2 in control and Par6b-depleted embryos. 50 ng of Par6b-MO was injected into the *crb3-gfp* or *gfp-lgl2* injected host transfer embryos. Western blot analysis at st12.5 and 17 with anti-GFP revealed two Crb3-GFP bands migrating at 50 kD and 35 kD, respectively. The 50 kD corresponds to the predicted size of full length Crb3-GFP, whilst the 35 kD band appears to be a partial C-terminal fragment with GFP. Par6b depletion caused an increase of the 50 kD Crb3-GFP band and a corresponding decrease of the 35 kD band at both stages (Fig. 6F), suggesting depletion of Par6b stabilizes the full-length Crb3-GFP. We also found that Par6b depletion caused modest but reproducible reduction of the GFP-Lgl2 protein level (Fig. 6F), consistent with the diminished GFP-Lgl2 immunofluorescense.

These data suggest that Par6b regulates apicobasal polarity in the developing epidermis by regulating Crb3 and Lgl2 protein stability. Par6b depletion reduces Lgl2 in both the superficial and deep layers, whilst Crb3 becomes inappropriately stabilized to the entire deep cell surfaces. In effect Parb6 depletion results in a loss of “basolateral” identity in both deep and superficial cells, with deep cells acquiring “apical” identity over the entire membrane. These data also explain the loss of E-cadherin, which is specifically localized to basolateral membranes of the epidermis (Fig. 4A, A′) [41], [43].

## Discussion

**Figure 7:**
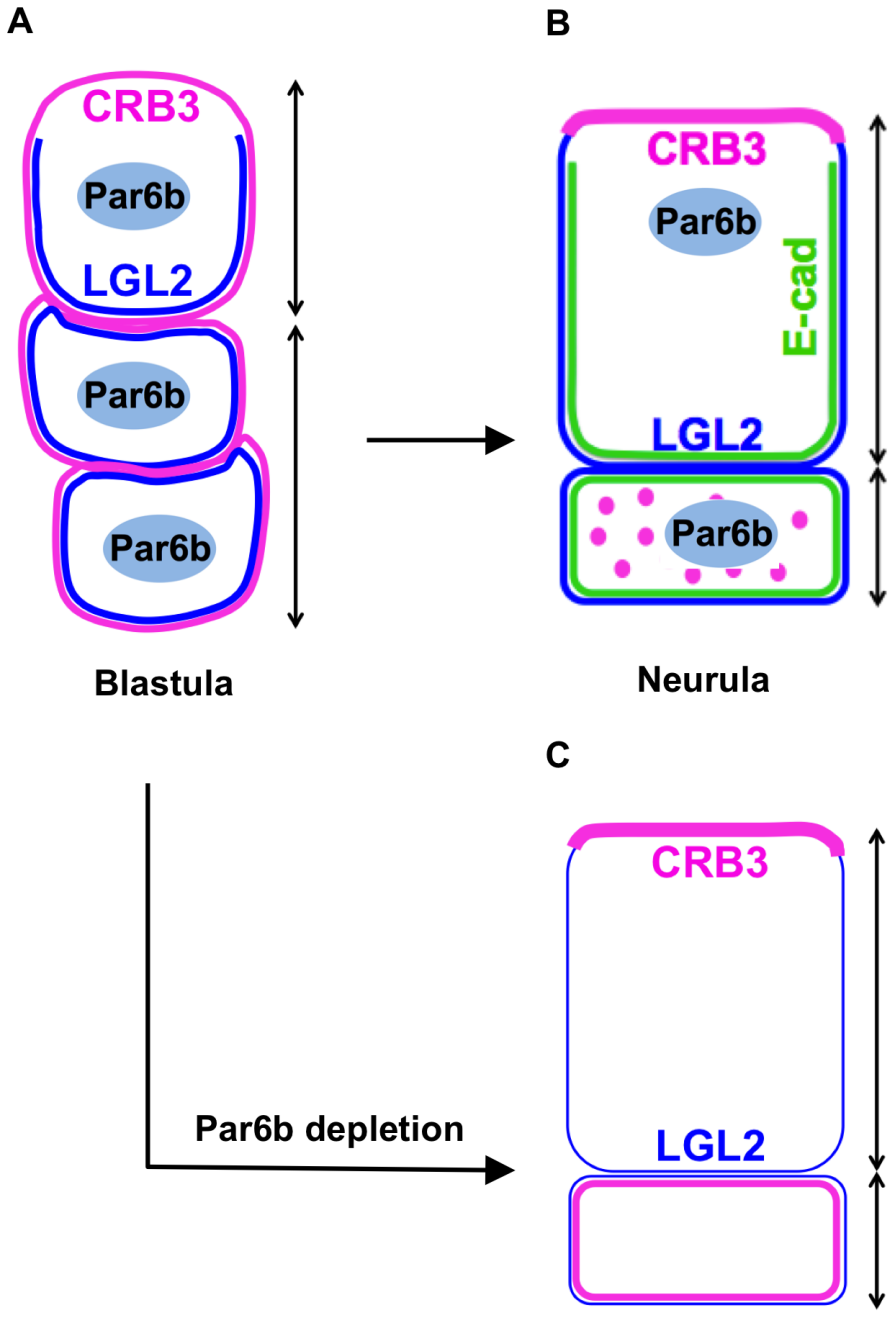
Summary model of the role of Par6b in developing stratified *Xenopus* epidermis. (**A**) The blastula presumptive epidermis. Crb3 is all around membranes of both superficial and deep cells. Lgl2 is basolaterally restricted in superficial cells and evenly distributed on all membranes of deep cells. (**B**) The neurula differentiated epidermis. Crb3 is apically restricted in superficial cells whilst forms cytoplasmic punctae in deep cells. Lgl2 is basolaterally restricted in superficial cells and on all membranes of deep cells. E-cadherin has similar distribution pattern as Lgl2. (**C**) The Par6b-depleted neurula epidermis. Superficial cells have Crb3 on apical membrane but less Lgl2 on basolateral membrane. Deep cells have both Crb3 and Lgl2 evenly distributed all around the membrane. E-cadherin is lost in both layers. (**A–C**) Double-headed arrows on the right side of each panal indicate boundaries of the superficial and deep layers.

In this study we show that apicobasal polarity in developing stratified epidermis of *Xenopus* is dynamically established during gastrulation and that Par6b regulates this process. Initially the blastula presumptive epidermal cells have an intermediate polarity with Crb3 around the entire membranes of both the superficial and deep cells, whereas Lgl2 is basolaterally restricted in the superficial cells but all around the entire deep cells surface (Fig. 7A). As gastrulation proceeds, the superficial cells become fully polarized with mutually exclusive Crb3+ apical and Lgl2+ basolateral membrane domains. In the deep cells, Crb3 is progressively lost from the membrane and sequestered into cytoplasmic particles, whilst Lgl2 is retained around the entire deep cell surface (Fig. 7B). This suggests that the deep cell membranes have a “basolateral” identity. We show that Par6b plays an essential role in this polarity remodeling process in both superficial and deep layers. Without Par6b, epidermal cells are less basolateralized with reduced expression of Lgl2 in both layers and Crb3 inappropriately localized around the entire deep cell surfaces (Fig. 7C). This is accompanied by a loss of basolaterally localized cadherin proteins (Fig. 7C) and subsequent sloughing of epidermal cells from Par6b-depleted embryos. We conclude that Par6b inhibits apical and promotes basolateral membrane identities and is required for epidermal integrity.

### The Apicobasal Polarity of the Developing *Xenopus* Stratified Epidermis

At the cleavage stage, *Xenopus* animal blastomeres divide asymmetrically and generate polarized superficial cells and non-polarized deep cells [23], [44]. The non-neural (presumptive epidermal) superficial ectodermal cells go through three differentiation states: undifferentiated (st9–12), intermediate (st13–16), fully differentiated (st17 onwards) epithelial cells. As differentiation proceeds, cytological organization become polarized along the apicobasal axis at st13 [32]. Our data show that superficial cells have already finished the segregation of Crb3 and lgl2 into two distinct membrane domains by st12. This cell membrane regionalization happens earlier than the cytoplasm polarization [32], which suggests that membrane polarization could be a pre-requisite for cytoplasmic components to break their symmetry. Interestingly, although deep cells do not possess physical boundaries TJs separating apical and basolateral membrane domains, their membranes acquire basolateral identity by excluding the apical marker Crb3 during development. In addition, we characterize a time window (st10.5–12) of apicobasal polarity remodeling process, which helps us to identify the correct order of apicobasal polarity establishment in further study.

### The Role of Par6b in Apicobasal Polarity of Stratified Epithelia

The *in vivo* role of Par6 in epithelial polarization has been studied in *C.elegans* [6] and *Drosophila* [7], they but leads to opposite conclusions. In *C.elegans*, Par6 is not essential for epithelial polarity. When both maternal and zygotic Par6 are removed, apical cytoskeletal markers such as microtubules, apical proteins such as Par3, and the basolateral protein LET-413 are all still asymmetrically localized in embryonic epithelial cells [6]. However, when Par6 is absent in *Drosophila*, epithelial cells become round and irregularly arranged, and apical proteins armadillo and Par3 fail to localize, indicating that Par6 is required for epithelial polarity in *Drosophila* [7]. These two studies were carried out in non-vertebrate simple epithelia, leaving the role of Par6 in stratified epidermis an open question. Here we demonstrate that Par6b is required for apicobasal polarity of stratified epidermis in *Xenopus*. Different requirements for Par6 among these three species could be due to differences in epithelial structure (simple versus stratified epithelia, non-vertebrate versus vertebrate), or because *Xenopus* has two Par6 proteins, Par6b and Par6g, but *C. elegans* and *Drosophila* only have one Par6. These differences reflect an evolving role of Par6 in epithelial apicobasal polarity [6].

Crb3 is a transmembrane protein. Our work reveals that Crb3 exists in two forms: membrane versus cytoplasmic Crb3, or full-length versus partial fragment with C-terminus. We show that Par6b depletion reduced cytoplasmic Crb3 and stabilized membrane Crb3 in deep epidermal cells. This was accompanied by an increase in the amount of the full-length relative to the partial fragment of Crb3. These suggest that full-length Crb3 likely stays on membrane whilst the fragment Crb3 with C-terminus is in cytoplasm. Cleavage of Crb3 has not been reported and may represent a novel mechanism for apical polarity remodeling. Par6 can interact with Crb3 directly via its own PDZ domain [22] or indirectly by binding to PALS1 [45]. We postulate that Par6b might bind to Crb3, and facilitates its proteolytic cleavage by a yet to be identified protease, after which the cytoplasmic domain of Crb3 is internalized, forming cytoplasmic punctae in deep cells. Alternatively, Par6b may be essential for endocytic trafficking of Crb3, after internalization, Crb3 is cleaved. We also show that Par6b depletion destabilized Lgl2 in both layers. Par6 can bind Lgl and allow aPKC-induced Lgl phosphorylation, which causes Lgl to accumulate on basolateral membranes [26], [46]. It is possible that, without Par6b, Lgl2 can’t be phosphorylated and is not stable on basolateral membranes. Alternatively, Par6b might bias vesicle trafficking toward the basolateral rather than apical membrane. Further experiments are required to test these possible mechanisms.

### Par6b and Adherens Junctions (AJs)

It has been suggested that AJs maturation depends on apical and basolateral cues [47], but how apicobasal polarity complexes regulate AJs is not fully understood. Par6 is required for junction formations in fly and worm epithelial cells [7], [16]. However, *in vitro* inhibition of Par6b in the breast cancer cell line MCF7 does not affect AJs formation [48]. It is not clear whether these differences are due to different AJs properties between non-chordates and chordates or different tissue contexts. Our study shows that Par6b is required to maintain AJs in the *Xenopus* epidermis through mechanism involving E-cadherin regulation. E-cadherin is first detectable at late gastrula stage when polarity of both layers is established [43], and it is restricted to basolateral membranes, overlapped with Lgl2 [41], [43]. Lgl2 can interact with myosin II heavy chain and the basolateral targeted syntaxin 4, suggesting its potential role in trafficking proteins like E-cadherin to the basolateral membrane [25]. Thus loss of Lgl2 might explain the reduced membrane E-cadherin in Par6b-depleted epidermis. Consistent with this interpretation, E-cadherin protein is destabilized at AJs in *lgl2* mutant zebrafish epidermis, without changes in mRNA levels [49].

In conclusion, our results reveal that Par6b regulates not only the dynamics of apicobasal polarity but also integrity of the developing stratified epidermis. These findings are important for three reasons. First, we characterize the apicobasal polarity identity in all layers of the developing stratified epidermis, which was previously unknown. Second, we show that Par6b regulates apicobasal polarity by repressing “apical” and promoting “basolateral” membrane identity. Third, we show that Par6b is required *in vivo* for proper E-cadherin expression at AJs, an observation that was controversial [7], [16], [48].

## Materials and Methods

### Ethics Statement

All *Xenopus laevis* experiments in this study have been conducted under protocol # 2D02014, approved by the Cincinnati Children’s Research Foundation’s Institutional Animal Care and Use Committee.

### Oocytes and Embryos

All *Xenopus laevis* animals used in this study were purchased through Nasco (Fort Atkinson, WI). Mature (st VI) oocytes used in host transfer experiments were obtained by manual defolliculation from ovaries of 2-year-old female frogs. Defolliculated oocytes were cultured in oocyte culture medium (OCM) and microinjected with mRNA five hours before maturing, and they were fertilized using the host transfer method [34]. Embryos were cultured in 0.1×MMR and dejellied in a 2% cysteine solution in 0.1×MMR (pH 7.8). For microinjection of mRNA and/or morpholino oligos at the 8- or 16-cell stage, embryos were transferred to 2% Ficoll solution in 0.5×MMR. Morpholino oligos were injected together with Rhodamine lysine dextran (RLDX) or GFP mRNA as lineage tracers. In MO+mRNA rescue experiments, morpholino was injected first followed by subsequent injection of the mRNA into the same cell.

### DNA Constructs, mRNAs and Oligonucleotides

*crb3-gfp* and *gfp-lgl2* mRNA were synthesized using constructs kindly provided by Nancy Papalopulu. A *Xenopus tropicalis* (*Xt) crb3* clone (Tegg038L10) was identified using ESTs and the coding sequence (GenBank AY884237) cloned into pCS2. An *Xt lgl2* clone was identified (Tegg006o20) from the ESTs, sequenced (GenBank AY884236) and the coding sequence cloned into pCS2. A pCS2 *gfp* construct was made for mRNA overexpression and producing fusion proteins by cloning the coding sequence of GFP into pCS2. *gfp-lgl2* and *crb3-gfp* were made by cloning the coding sequence of each protein into pCS2 *gfp* [23]. Full-length *Xenopus laevis par6b* cDNA was acquired from *Xenopus laevis* genomic cDNA. 1-HA tagged *par6b* was cloned by proofreading PCR amplification. Capped mRNA was synthesized using the Sp6 mMESSAGE mMACHINE Kit.

5′ *Xl par6b* BamHI ATG primer:

5′-GCGGATCCAATATGAACCGGGGACACCGGG –3′

3′ *Xl par6b* XhoI in frame to pCS2+HA

5′-CGCTCGAGCAGTGTAAGCACGGTACCGT –3′

*Xl* Par6b-MO (translational blocking):

5′-GTCCCCGGTTCATGTTGCCAGTGCA-3′ [29].

Control-MO: 5′-CCTCTTACCTCAGTTACAATTTATA-3′. And antisense morpholinos were purchased through Gene tools, LLC.

### Quantitative RT-PCR and *in situ* Hybridization

Total RNAs from embryos was isolated using the protocol of Mir et al. [50]. Real-time RT-PCR was performed using a LightCycler (Roche). Water-blank and RT-minus controls were included in all runs. All RT-PCR results are presented as percentages compared with the levels in uninjected embryos after normalization to the expression of ornithine decarboxylase (*odc*). Embryos were cultured and processed for whole-mount ISH using a protocol described previously [51]. Sense and antisense *par6b* Dig-labeled RNA probes were synthesized using Ambion MEGA script kits and digoxygenin-11-UTP.

### Western Blotting

Batches of five embryos were homogenized in 50 µl of ice-cold PBS-triton (PBS+1% Triton-x100) plus protease inhibitors (PIC P8340 Sigma 1∶100, PMSF 10 µg/ml) and spun for 5 min first at 800 g and then 12000 revolutions per min at 4°C. Clear lysates were diluted in equal volumes of 2 × Laemmli Sample buffer (BIO-RAD) and boiled for 5 min. A total of 0.5 to 1 embryo equivalent was loaded onto a 10% Tris-glycine-SDS gel. Electrophoresis, electro transfer, and antibody staining were performed as described previously [52]. Primary antibodies were: mouse anti-E-cadherin (5D3 monoclonal, DSHB, 1∶3000 dilution), mouse anti-C-cadherin (6B6 monoclonal, DSHB, 1∶1000 dilution), mouse anti-tubulin (DM1A, Neomarker, 1∶10000 dilution), and mouse anti-GFP (sc-9996, Santa Cruz biotechnology, 1∶3000 dilution).

### Immunofluorescence

For Crb3-GFP and GFP-Lgl2 imaging, *crb3-gfp* or *gfp-lgl2* mRNA-injected host transfer embryos were fixed in MEMFA for 1.5 to 2 hours at room temperature. Fixed embryos were embedded in 4% low-melting agarose for vibratome sectioning and sectioned at 100 µm thickness. Sectioned samples were mounted in 1×PBS and imaged under FITC excitation using a Nikon A1Rsi inverted confocal microscope. A LD C-Apochromat 40×/1.1 W korr UV-vis IR objective was used for high magnification. For immunofluorescence, *Xenopus* embryos were fixed in trichloroacetic acid (TCA) for 3 hours at RT or MEMFA overnight at 4°C. The following antibodies were used, mouse anti-E-cadherin (5D3 monoclonal, DSHB, 1∶200 dilution), mouse anti-C-cadherin (6B6 monoclonal, DSHB, 1∶200 dilution), mouse anti-tubulin (DM1A, Neomarker, 1∶500 dilution), and mouse anti-GFP (sc-9996, Santa Cruz biotechnology, 1∶200 dilution), rabbit anti-GFP (sc-8334, Santa Cruz biotechnology, 1∶200 dilution), Mouse anti-β1 integrin (8C8 monoclonal, DSHB, 1∶200 dilution), rabbit anti-active Caspase-3 (BD Biosciences, 1∶200 dilution).

## Supporting Information

Figure S1 **The expression pattern of** ***par6b*** **during development.** (**A**) Sagittal view of *par6b* in a st IV oocyte by whole-mount ISH. (**B, B’**) *par6b* expression pattern on a sagittal section of st9 embryo and the magnified view of the box area. (**C**) Lateral view of *par6b* expression pattern at st11. (**D, D’**) *par6b* expression pattern on a sagittal section of st11 embryo and the magnified view of the box area. (**E**) Lateral view (head toward the left) of *par6b* expression pattern at st24. (**F, F’**) *par6b* expression pattern on a sagittal section of st24 embryo and the magnified view of the box area.
(TIF)

Figure S2 **Control-MO injection does not change E- and C-cadherin expression.** (**A–B′**) Embryos were injected with Par6b-MO together with GFP into one animal ventral blastomere at the 8-cell stage. Staining of E-cadherin (A, A′) or C-cadherin (C, C′) (red) and GFP (green) on the section of st17 injected embryos.
(TIF)

Figure S3 **Par6b depletion causes reduction of other epidermal adhesion molecules without an elevation of apoptosis.** (**A–F**) Staining of β-, α- and γ- catenins, β1- and α5-integrins, and tight junction ZO-1 (red) respectively on sections of st17 epidermis with Par6b-MO injected clones (GFP positive, green). Scale bars, 50 µm.
(TIF)

Figure S4 **Par6b depletion does not change ectoderm cell fate.** (**A**) Quantitative RT-PCR assays of expression of ectodermal markers *epidermal keratin*, *sox2* and *n-cadherin* mRNA levels after two doses of Par6b-MO injection from the late blastula (st9) to neurula (st21) stage. (**B**) Cytokeratin staining on the transverse section of st17 embryos that contain Par6b-MO injected clones (GFP positive). Scale bars, 50 µm.
(TIF)
