# Supplementary figures and images for: Par6b Regulates the Dynamics of Apicobasal Polarity during Development of the Stratified Xenopus Epidermis
Source: PLoS One. 2013 Oct 18;8(10):e76854. doi: 10.1371/journal.pone.0076854 (PMC3800127; doi:10.1371/journal.pone.0076854)

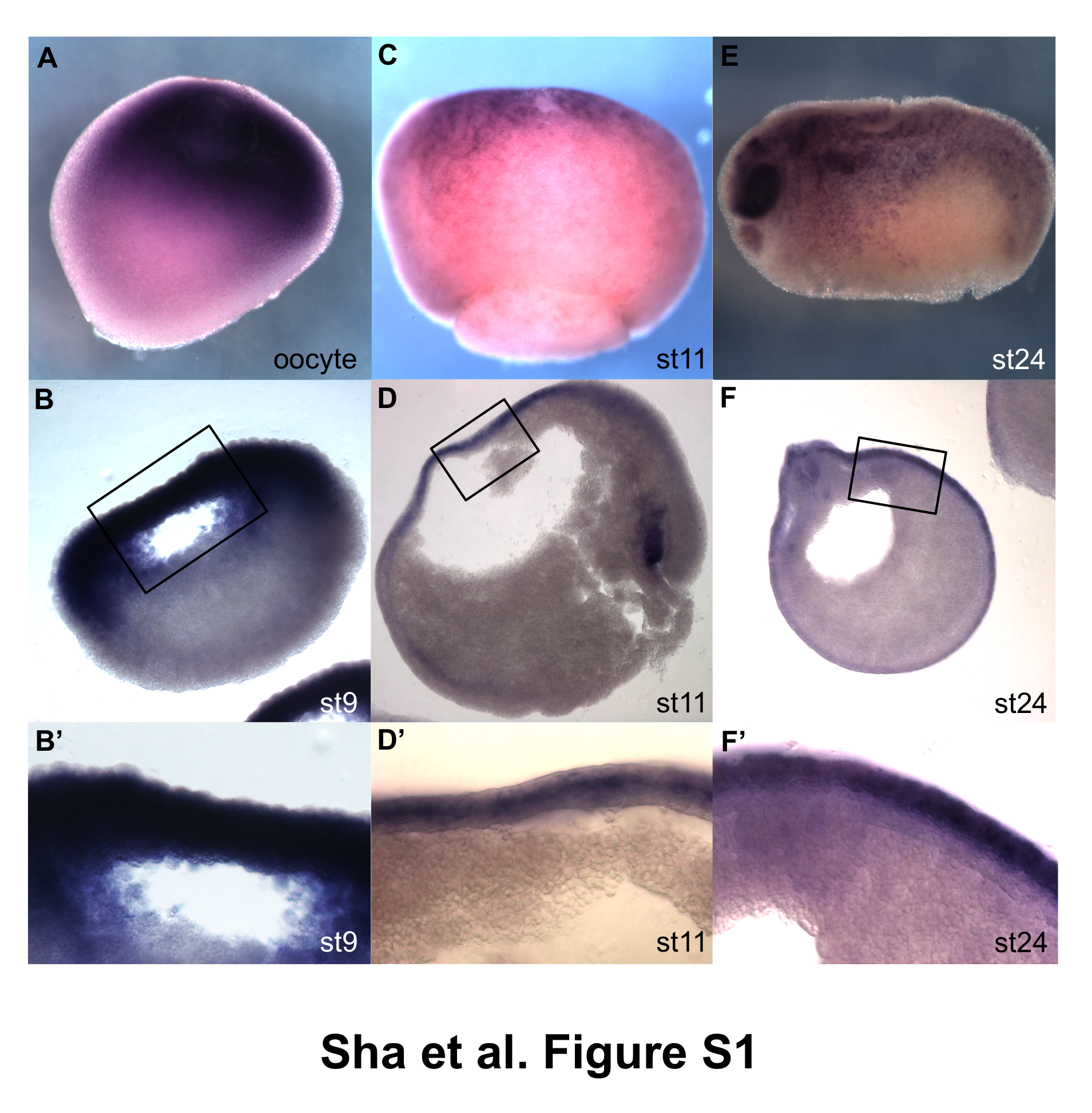

Supplement: Figure S1 — The expression pattern of par6b during development. (A) Sagittal view of par6b in a st IV oocyte by whole-mount ISH. (B, B’) par6b expression pattern on a sagittal section of st9 embryo and the magnified view of the box area. (C) Lateral view of par6b expression pattern at st11. (D, D’) par6b expression pattern on a sagittal section of st11 embryo and the magnified view of the box area. (E) Lateral view (head toward the left) of par6b expression pattern at st24. (F, F’) par6b expression pattern on a sagittal section of st24 embryo and the magnified view of the box area. (TIF) [file pone.0076854.s001.tif]

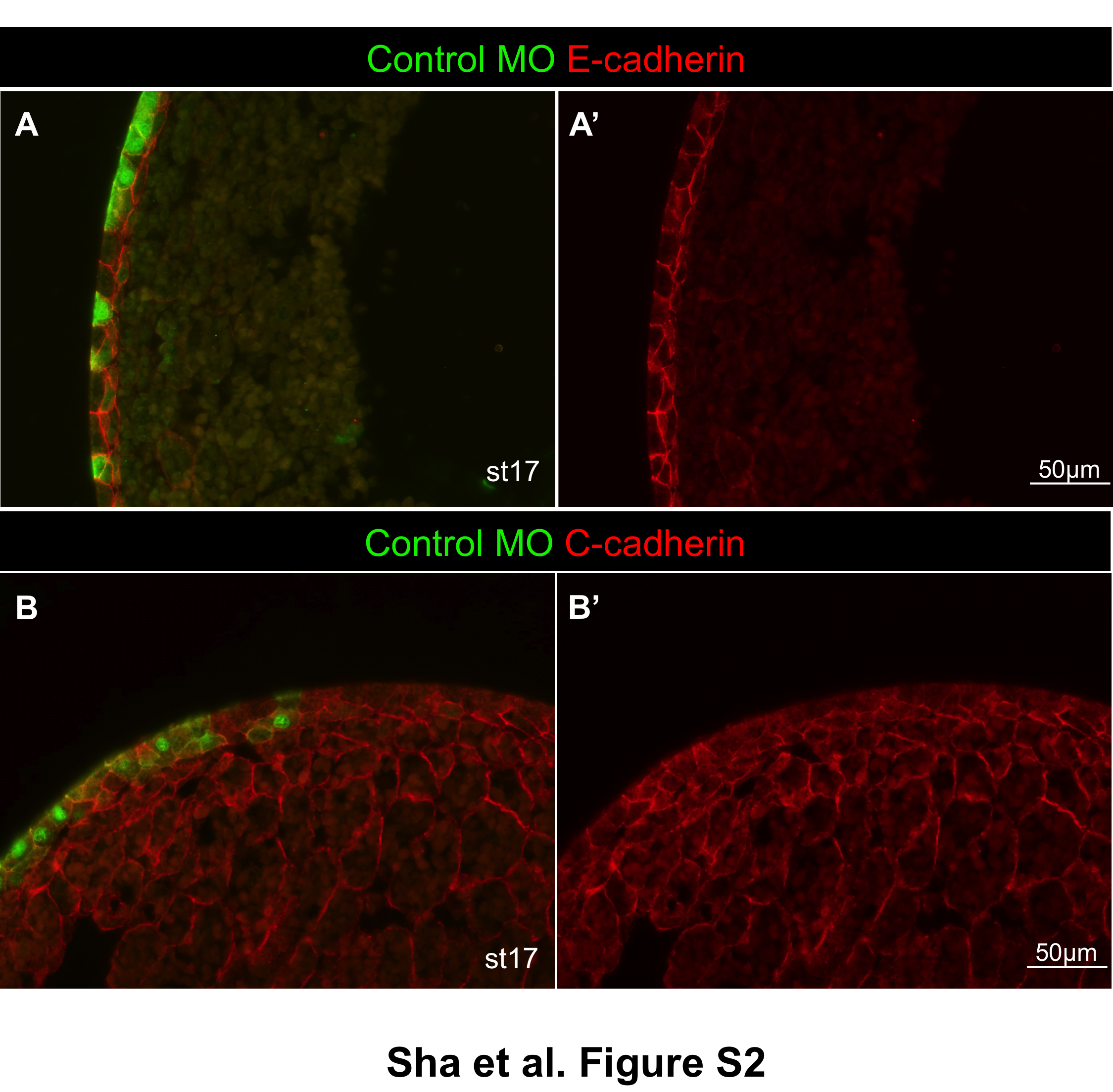

Supplement: Figure S2 — Control-MO injection does not change E- and C-cadherin expression. (A–B′) Embryos were injected with Par6b-MO together with GFP into one animal ventral blastomere at the 8-cell stage. Staining of E-cadherin (A, A′) or C-cadherin (C, C′) (red) and GFP (green) on the section of st17 injected embryos. (TIF) [file pone.0076854.s002.tif]

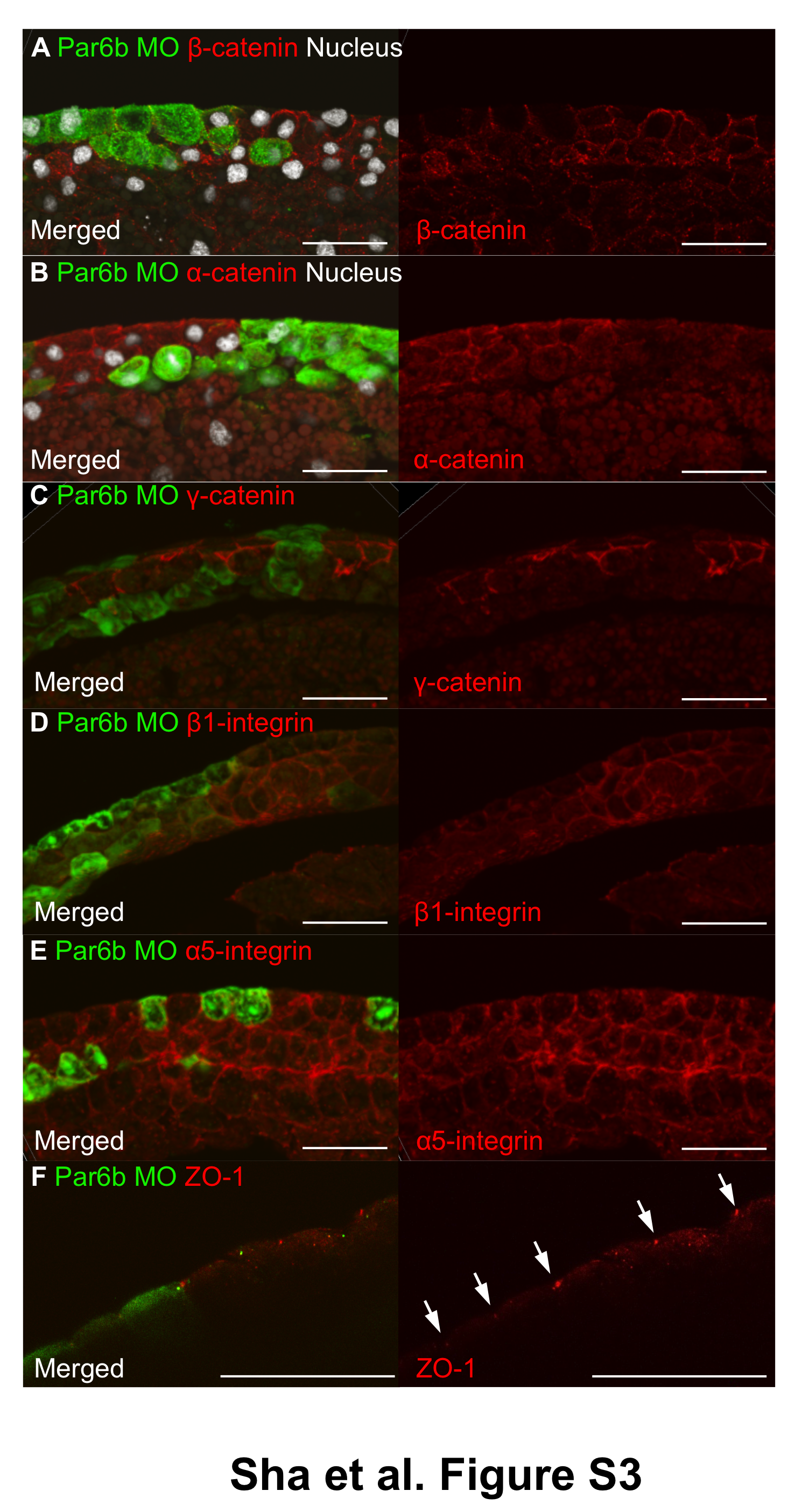

Supplement: Figure S3 — Par6b depletion causes reduction of other epidermal adhesion molecules without an elevation of apoptosis. (A–F) Staining of β-, α- and γ- catenins, β1- and α5-integrins, and tight junction ZO-1 (red) respectively on sections of st17 epidermis with Par6b-MO injected clones (GFP positive, green). Scale bars, 50 µm. (TIF) [file pone.0076854.s003.tif]

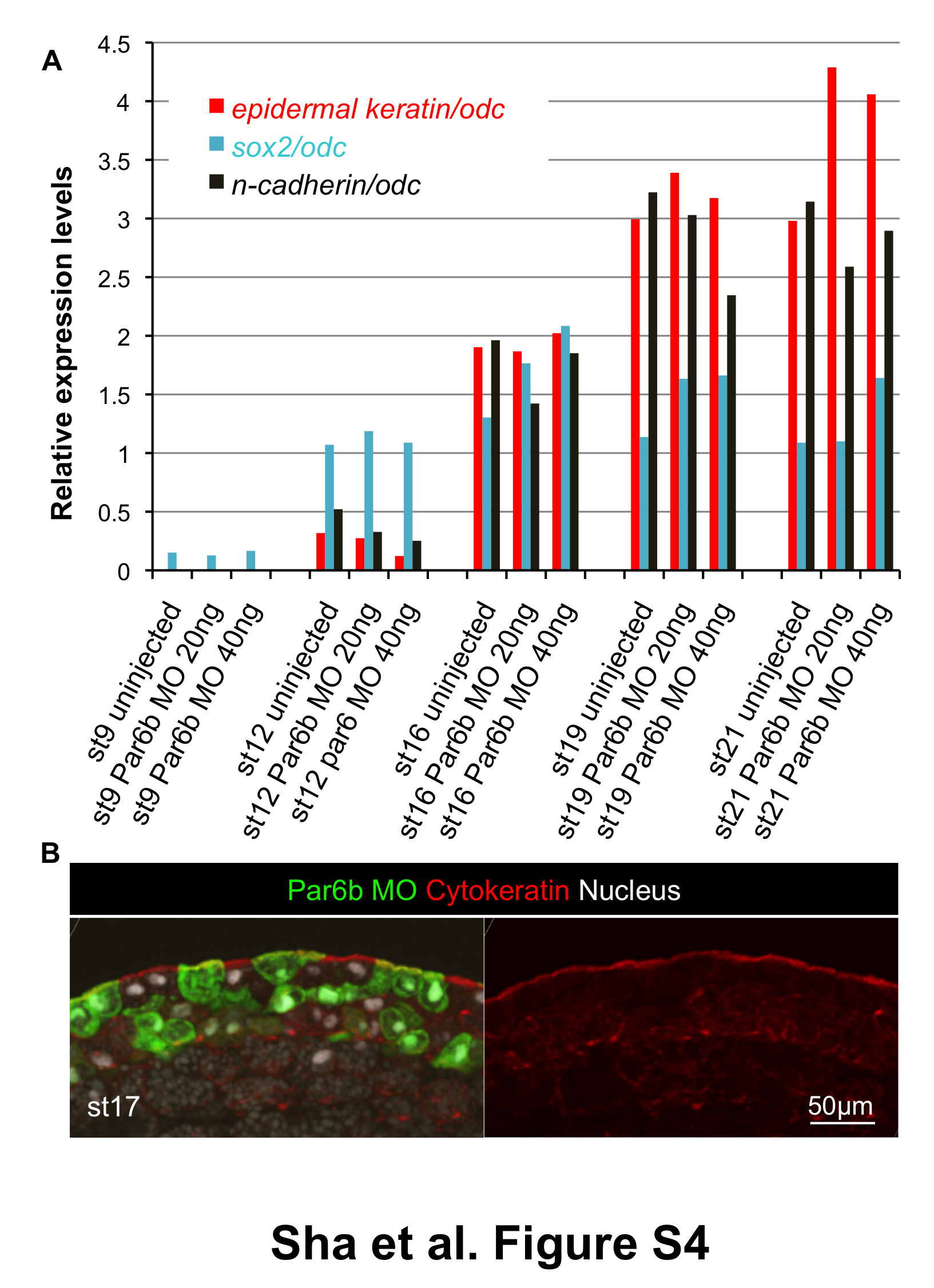

Supplement: Figure S4 — Par6b depletion does not change ectoderm cell fate. (A) Quantitative RT-PCR assays of expression of ectodermal markers epidermal keratin, sox2 and n-cadherin mRNA levels after two doses of Par6b-MO injection from the late blastula (st9) to neurula (st21) stage. (B) Cytokeratin staining on the transverse section of st17 embryos that contain Par6b-MO injected clones (GFP positive). Scale bars, 50 µm. (TIF) [file pone.0076854.s004.tif]
